# Epidemiological characteristics and prognostic risk factors of pulmonary blastoma based on the 2021 WHO classification

**DOI:** 10.1038/s41598-025-08571-5

**Published:** 2025-07-01

**Authors:** Lu Yang, Lei Yu, Shimin Tang, Ji Ma, Qiang Zhou, Li Liu, Yong Li, Na Li

**Affiliations:** 1 Suining Central Hospital, Suining, Sichuan China; 2 Department of Radiology and Imaging, Suining Central Hospital, Suining, Sichuan China; 3 Department of oncology, Suining Central Hospital, Suining, Sichuan China

**Keywords:** Pulmonary Blastoma, Epidemiological characteristics, Risk factors, Rare lung malignancy, Non-small-cell lung cancer, Cancer epidemiology, Cancer

## Abstract

Pulmonary blastoma (PB) is a rare and aggressive lung malignancy. Prior studies were confounded by inconsistent histological classifications, particularly before the 2021 World Health Organization (WHO) reclassification. This study aimed to characterize PB’s epidemiological features and prognostic factors under the updated 2021 WHO criteria. Data on patients diagnosed with PB from the The Surveillance, Epidemiology, and End Results (SEER) database between 2000 and 2020 were collected. Kaplan-Meier curves were used to evaluate overall survival (OS), and univariate/multivariate Cox regression analysis was employed to identify independent factors affecting prognosis. PB affected females more frequently (61.5%) and occurred across all ages without a clear peak. The median tumor size was 77.83 mm, with upper/lower lung lobes being common sites. Surgical resection was performed in 73.8% of patients. The 1-, 3-, and 5-year OS rates were 70.3%, 57.2%, and 51.9%, respectively. Multivariate analysis revealed age, histological grade, and surgical status as independent prognostic factors. This study provides the first comprehensive analysis of PB under the 2021 WHO classification, demonstrating improved survival outcomes compared to historical reports. Age, histological grade, and surgical intervention are critical prognostic determinants. These findings underscore the importance of accurate histological classification and surgical resection in optimizing PB management.

## Introduction

Lung cancer, a significant component of the global cancer burden, presents substantial challenges to clinical management due to its diverse pathological subtypes and inherent complexity. Pulmonary blastoma (PB), a rare subtype of lung cancer, constitutes approximately 0.1% of all pulmonary tumors. Initially described by Barnett and Barnard in 1945, it was termed “pulmonary fetal tissue tumor” due to its histological resemblance to the lung tissue of a 3-month-old fetus^1,2^. Historically, “pulmonary blastoma” encompassed several distinct tumor types, including classic biphasic PB (CBPB), well-differentiated fetal adenocarcinoma (WDFA), and pleuropulmonary blastoma (PPB)^3^. However, the 2021 WHO classification marked a critical advance by explicitly distinguishing PB from PPB and WDFA as distinct entities: PB (encompassing classic biphasic subtype, CBPB) was reclassified as a sarcomatoid carcinoma, PPB as a mesenchymal tumor, and WDFA as a subtype of adenocarcinoma^4,5^. This revision addresses historical ambiguities in previous studies^6^, which conflated these entities, leading to biased survival outcomes and prognostic conclusions.

Due to the rarity of pulmonary blastoma, epidemiological data are scarce and fragmented. It is estimated that fewer than a hundred new cases of PB are diagnosed globally each year. The principal challenges in epidemiological research include difficulties in case acquisition, variability in diagnostic criteria, and a dearth of long-term follow-up data. The Surveillance, Epidemiology, and End Results (SEER) program, a population-based cancer registry system in the United States, encompasses data from approximately 48% of the U.S. population, encompassing epidemiological characteristics, clinical information, survival status, and follow-up duration. Recognizing the rarity of PB and its profound impact on patient survival, a thorough investigation into its epidemiological traits and risk factors is essential for scientific inquiry and urgently needed to enhance early detection rates, refine treatment protocols, and ameliorate patient outcomes. Given the lack of large-scale studies exclusively focusing on PB under the 2021 classification, this study utilizes the SEER database to analyze the epidemiological features and prognostic risk factors of PB. By excluding PPB and WDFA cases, we aim to provide the first comprehensive insights into PB as an independent entity, thereby refining clinical strategies and addressing gaps in prior research.

## Materials and methods

### Data sources

This study is based on the SEER database released in November 2022. First, access to the SEER database was obtained, and data was collected using the SEER*Stat software (version 8.4.2).

Inclusion criteria: Malignant tumors diagnosed between 2000 and 2020 with ICD-O-3 Hist/Behav codes 8972/3 (pulmonary blastoma). (2) Exclusion criteria: Patients diagnosed by autopsy or death certificate. Variables collected: Age, gender, race, marital status, year of diagnosis, tumor size, primary site, laterality, pathological grade, treatment methods (surgery, radiotherapy, chemotherapy), survival status, and survival time.

### Statistical analysis

This study utilized SPSS 26.0, R software (version 4.3.1), and X-title (http://tissuearray.org, version 3.6.1) for statistical analysis. Categorical variables are represented by frequency and percentage, while continuous variables that conform to a normal distribution are presented as “Mean ± SD”. X-title software was employed to identify the optimal cutoff value for tumor size. Kaplan-Meier survival curves were used to assess the differences in patient survival rates. Furthermore, univariate and multivariate Cox regression analyses were applied to determine the independent prognostic factors affecting OS. All statistical tests were conducted as two-tailed, with a P-value less than 0.05 considered statistically significant.

## Results

### Demographic and clinical pathological characteristics

In this study, we analyzed data from 65 patients diagnosed with PB between 2000 and 2020. The majority of these patients were Caucasian, representing 75.38% of the study population. There was a notable gender disparity among PB patients, with females constituting 61.54% of the cases, which is 1.6 times the number of male patients. Regarding age at onset, PB did not exhibit a distinct pattern, though there was a slightly higher prevalence among younger patients compared to the elderly. In terms of tumor laterality, there was a slight preponderance for the right lung (41.54% vs. 53.85%). The upper and lower lobes were the most frequent tumor sites, with additional cases occurring in the main bronchus, middle lobe, and as overlapping lesions within the lung lobes. While the pathological grading for over half of the PB patients was indeterminate, among those with known grades (36.92%), a majority had a grade I or II. Surgical intervention was the predominant treatment approach, with 73.85% of patients receiving surgery. Chemotherapy was administered to 43.08% of patients, and radiotherapy was utilized in only 26.15% of cases (Table 1).

**Table 1:** Baseline characteristics of the 65 pulmonary Blastoma cases.

Variables	Total (*n* = 65)
Marital, n(%)	
Married	25 (38.46)
Single	37 (56.92)
Unknown	3 (4.62)
Race, n(%)	
White	49 (75.38)
Black	14 (21.54)
Other	2 (3.08)
Gender, n(%)	
Female	40 (61.54)
Male	25 (38.46)
Age, n(%)	
00 years	1 (1.54)
01–04 years	6 (9.23)
15–19 years	2 (3.08)
25–29 years	5 (7.69)
30–34 years	1 (1.54)
35–39 years	5 (7.69)
40–44 years	5 (7.69)
45–49 years	5 (7.69)
50–54 years	5 (7.69)
55–59 years	4 (6.15)
60–64 years	5 (7.69)
65–69 years	3 (4.62)
70–74 years	5 (7.69)
75–79 years	6 (9.23)
80–84 years	4 (6.15)
85 + years	3 (4.62)
Primary Site, n(%)	
Main bronchus	5 (7.69)
Upper lobe, lung	22 (33.85)
Middle lobe, lung	3 (4.62)
Lower lobe, lung	23 (35.38)
Overlapping lesion of lung	3 (4.62)
Lung, NOS	9 (13.85)
Laterality, n(%)	
Bilateral, single primary	3 (4.62)
Left	27 (41.54)
Right	35 (53.85)
Tumor size, Mean ± SD (mm)	77.83 ± 44.41
Grade, n(%)	
I	12 (18.46)
II	2 (3.08)
III	8 (12.31)
IV	2 (3.08)
Unknown	41 (63.08)
Surgery, n(%)	
None	17 (26.15)
Yes	48 (73.85)
Radiation, n(%)	
None/Unknown	48 (73.85)
Yes	17 (26.15)
Chemotherapy, n(%)	
No/Unknown	37 (56.92)
Yes	28 (43.08)

NOS: Not Otherwise Specified

### KM survival analysis

**Fig. 1:**
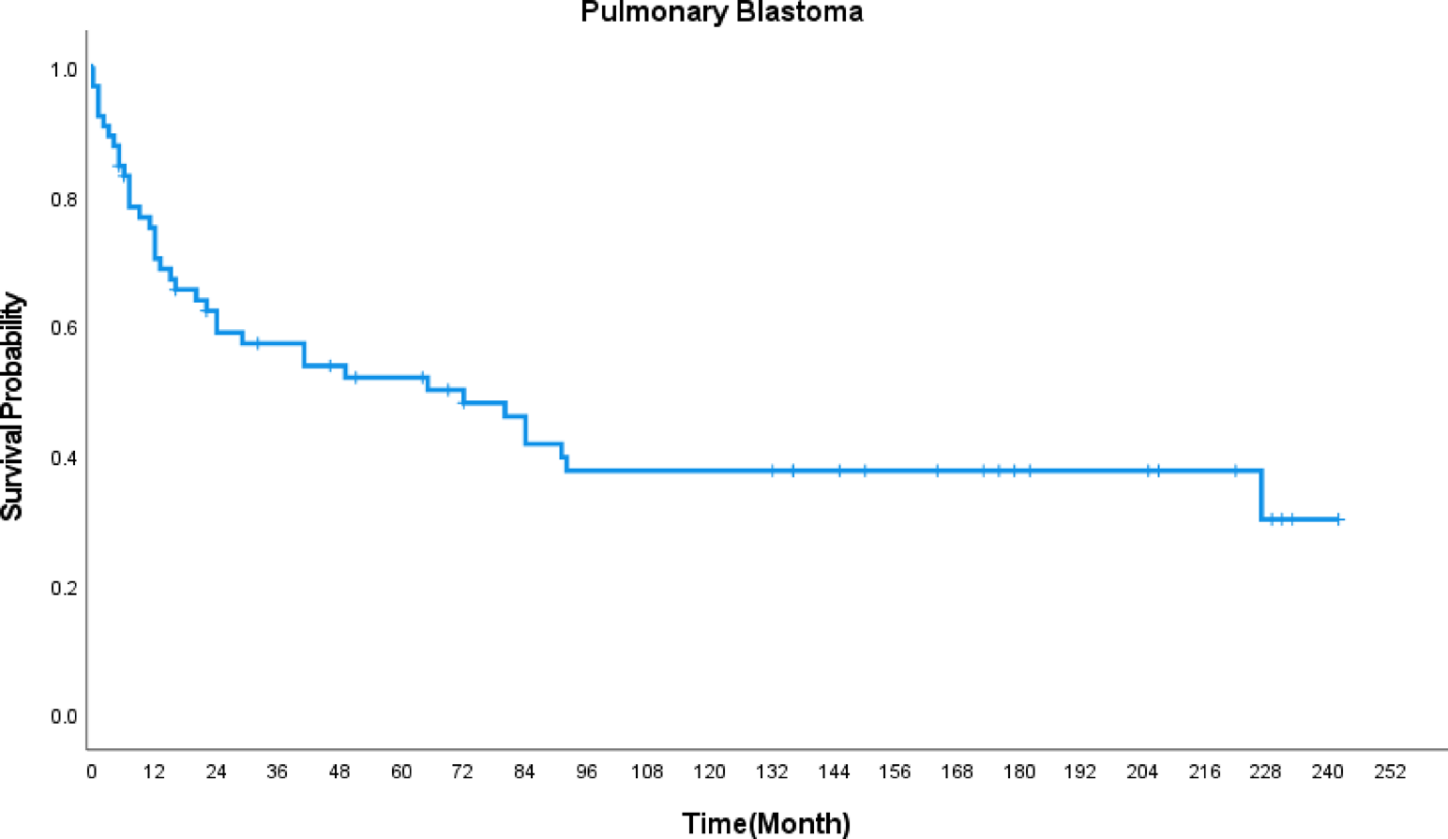
The Kaplan-Meier survival curve of PB patients.

**Fig. 2:**
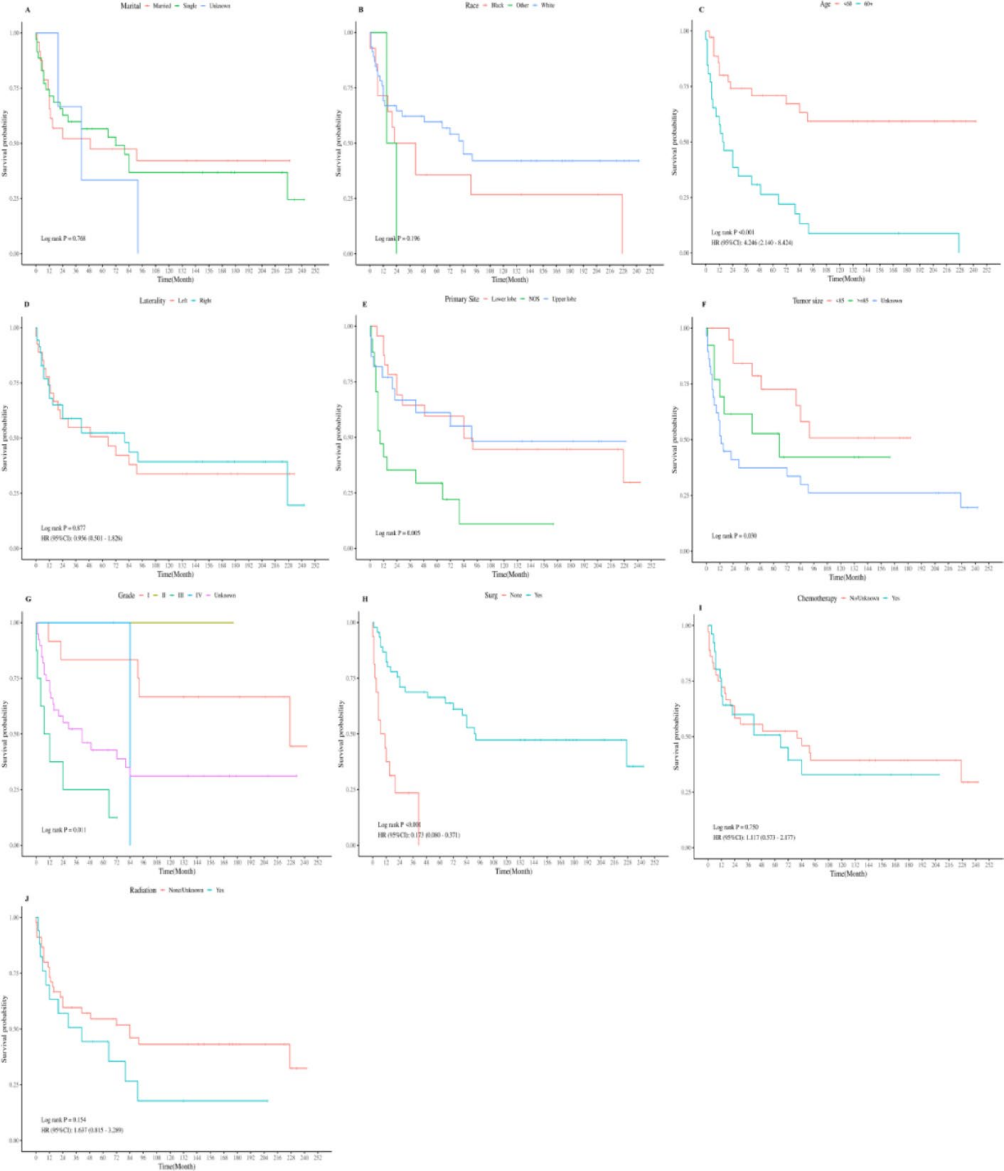
The Kaplan-Meier survival curve of PB patients under different classification conditions.

The 1-year OS of PB patients was 70.3%, the 3-year OS was 57.2%, and the 5-year OS was 51.9%. The median survival duration was 65 months (Fig. 1). X-title software determined the optimal cutoff value of tumor size for PB patients as 85 mm, and KM survival analysis showed that age, tumor primary tumor, tumor size, tumor histological pathology grade, and surgery are factors affecting the OS of PB patients (*P* < 0.05) (Fig. 2).

### Prognostic risk factors for PB patients

We incorporated a range of variables, including marital status, race, age, gender, tumor laterality, tumor primary site, tumor size, pathological grade, surgical intervention, radiotherapy, and chemotherapy, into both univariate and multivariate Cox regression models to identify the independent prognostic factors for PB patients. The univariate Cox regression analysis revealed that age, histological grade, primary site, tumor size, and surgical treatment were significantly associated with overall survival (OS) in PB patients. Further multivariate Cox regression analysis confirmed that age, histological grade, and surgical treatment are independent prognostic determinants for PB patients (Table 2).

**Table 2:** Prognostic factors for patients with pulmonary blastoma.

Variables	Univariate analysis		Multivariate analysis	
	*P*	HR (95%CI)	*P*	HR (95%CI)
Marital				
Married		1.00 (Reference)		
Single	0.894	1.05 (0.52 ~ 2.10)		
Unknown	0.480	1.57 (0.45 ~ 5.54)		
Race				
Black		1.00 (Reference)		
Other	0.534	1.62 (0.35 ~ 7.44)		
White	0.174	0.61 (0.30 ~ 1.25)		
Age				
<60		1.00 (Reference)		1.00 (Reference)
60+	< 0.001	4.21 (2.12 ~ 8.35)	< 0.001	4.24 (2.04 ~ 8.85)
Sex				
Female		1.00 (Reference)		
Male	0.055	1.89 (0.99 ~ 3.61)		
Grade				
I		1.00 (Reference)		1.00 (Reference)
II	0.998	0.00 (0.00 ~ Inf)	0.998	0.00 (0.00 ~ Inf)
III	0.002	6.85 (2.05 ~ 22.91)	< 0.001	12.83 (3.16 ~ 52.07)
IV	0.675	1.59 (0.18 ~ 13.88)	0.344	3.04 (0.30 ~ 30.55)
Unknown	0.036	2.88 (1.07 ~ 7.76)	0.055	2.99 (0.98 ~ 9.12)
Primary Site				
Lower lobe		1.00 (Reference)		
Upper lobe	0.979	1.01 (0.44 ~ 2.31)		
NOS	0.006	2.98 (1.36 ~ 6.53)		
Tumor size(mm)				
<85		1.00 (Reference)		1.00 (Reference)
>=85	0.260	1.79 (0.65 ~ 4.96)	0.660	1.27 (0.44 ~ 3.71)
Unknown	0.012	2.84 (1.25 ~ 6.45)	0.002	4.33 (1.70 ~ 11.03)
Laterality				
Left		1.00 (Reference)		
Right	0.878	0.95 (0.50 ~ 1.82)		
Surgery				
None		1.00 (Reference)		1.00 (Reference)
Yes	< 0.001	0.18 (0.08 ~ 0.38)	< 0.001	0.18 (0.08 ~ 0.44)
Radiation				
None/Unknown		1.00 (Reference)		
Yes	0.162	1.65 (0.82 ~ 3.31)		
Chemotherapy				
No/Unknown		1.00 (Reference)		
Yes	0.753	1.11 (0.57 ~ 2.17)		

HR: Hazard Ratio, CI: Confidence Interval

## Discussion

PB is a rare lung tumor. While Bu X et al.^6^ discussed the treatment and prognosis associated with PB in 2020, their study did not differentiate between PB and PPB. In contrast, the 2021 WHO classification system clearly delineates PB and PPB as distinct entities. PB is categorized as a sarcomatoid carcinoma, whereas PPB is recognized as a unique mesenchymal tumor. This distinction is based on the divergent histological features and clinical presentations observed in PB and PPB^4,5^.

This retrospective study analyzed data from 65 patients diagnosed with PB between 2000 and 2020, shedding light on the epidemiological characteristics and prognostic risk factors associated with this rare tumor. This analysis revealed a pronounced gender disparity among patients with PB, with females outnumbering males by a ratio of 1.6 to 1, corroborating the findings reported by Vossler JD et al. in 2019^7^. They proposed that this female preponderance may be associated with the overactivation of estrogen receptors by β-catenin. Similarly, Koss et al.^8^ reported a comparable male-to-female ratio, whereas Van Loo S et al.^9^ observed a higher incidence among male patients. This discrepancy may stem from previous studies’ failure to distinguish PB from PPB and WDFA. PB exhibits a broad age range at onset without a clear trend, and the tumors are typically unilateral and multiple, showing no significant laterality. Consistent with the studies by Koss MN^8^ and Van Loo S et al.^9^, the upper and lower lobes are the most common sites for PB. The average tumor size was measured at 77.83 ± 44.41 mm. Clinical symptoms of PB are non-specific, encompassing cough, hemoptysis, and chest pain, with approximately 40% of patients asymptomatic in the early stages^10^, often detected incidentally during chest X-ray examinations. PB is composed of an epithelial and mesenchymal stroma, with potential foci of chondrosarcoma, rhabdomyosarcoma, osteosarcoma, and yolk sac tumors^11^. Imaging plays a crucial role in diagnosing PB, typically presenting as a unilateral, round or oval solid mass with smooth margins and surrounding fissures, typically lacking ‘spiculation‘^12^. Surgical resection, including lobectomy, pneumonectomy, and lymph node dissection, forms the cornerstone of PB treatment. In this study, 73.85% of PB patients underwent surgery, which previous research indicates can offer long-term survival for patients with small, non-lymph node-involving tumors^13^. Additionally, radiotherapy and chemotherapy are integral to PB treatment, with 43.08% and 26.15% of patients receiving these therapies, respectively.

The prognostic outcomes of PB remain inconsistent across existing literature. In a study spanning 1977–1999, Robert J. et al.^14^ documented a median survival of 15 months (range: 6–30 months) among four PB patients, all of whom succumbed to the disease postoperatively. By contrast, Bu X et al.^6^,reported a more favorable 5-year OS rate of 58.2% based on a database analysis of cases from 1988 to 2016. Our cohort, however, demonstrated intermediate outcomes, with 1-year, 3-year, and 5-year OS rates of 70.3%, 57.2%, and 51.9%, respectively. The slightly worse prognosis observed in our study compared to Bu X et al.‘s^6^ findings may be attributable to methodological differences. Specifically, our analysis adhered to the updated 2021 WHO classification, which excluded the now-recognized independent entities, PPB and WDFA, while retaining CBPB - a subtype associated with poorer outcomes.Notably, prior studies indicate that two-thirds of CBPB patients decease within two years of diagnosis, with 5- and 10-year survival rates as low as 16% and 8%, respectively^15^. Additionally, our study encompassed cases from 2000 to 2020, during which advancements in early detection and therapeutic modalities may have contributed to modest improvements in PB prognosis overall.

Given the rarity of PB and the recent 2021 classification update, comprehensive big data analyses on prognostic risk factors are lacking. Our findings suggest that age, histological grade, primary tumor site, and surgical intervention are associated with OS in PB patients, with age above 60 years, poor histological grade, and absence of surgical treatment emerging as key predictive factors for a poor prognosis, aligning with the outcomes observed by Koss MN et al.^8^. Surgery is the principal treatment modality for PB, and radical surgical intervention can offer long-term survival for patients with small, non-lymph node-involved tumors^13^. Moreover, chemotherapy and radiotherapy are additional treatment options for PB patients. However, the efficacy of chemotherapy in PB remains uncertain, with some previous studies suggesting no benefit for PB patients^16–19^, Conversely, studies by Liu Q^20^ and Larsen H^21^ et al. have demonstrated that postoperative docetaxel and cisplatin can effectively manage recurrent PB and prolong patient survival. In our study, however, the prognostic benefits of radiotherapy and chemotherapy for PB were not significant. This may be attributed to the small sample size, which precludes further stratified analysis based on clinical staging to explore the important role of radiotherapy and chemotherapy in the management of PB. Beyond surgery, chemotherapy, and radiotherapy, innovative approaches are being explored for PB treatment. Chen Y et al.‘s^22^ study investigated the combination of immunotherapy with anti-angiogenic targeted therapy for PB, showing that the integration of immunotherapy and multi-target tyrosine kinase inhibitors may yield significant clinical benefits in PB and could potentially exhibit synergistic effects.

Despite providing new insights into the epidemiological characteristics and prognostic risk factors of PB, this study still has limitations. First, although the SEER database includes 48% of the U.S. population, due to the rarity of PPB, our study sample size is still limited, which may affect the generalizability of the results. Second, the SEER database is a national cancer statistics database in the United States, covering only specific regions and populations in the United States, which may have regional and population selection bias. In addition, the SEER database lacks some important prognostic-related characteristics, such as molecular markers and gene mutation status, which may have an important impact on the establishment and performance of the prognostic prediction model, but are difficult to obtain in the SEER database. Therefore, future studies need larger sample sizes and longer follow-up data to verify our findings and further explore the molecular mechanisms and treatment strategies of PB .

## Conclusion

This study provides initial evidence into PB, revealing a more favorable prognosis for patients when PB is classified according to the 2021 WHO criteria. With 1-year, 3-year, and 5-year overall survival rates of 70.3%, 57.2%, and 51.9% respectively. We identified age, histological grade, and surgical intervention as significant prognostic factors. Though the impact of chemotherapy and radiotherapy is not definitively established, our research hints at their potential role in managing recurrent cases. The epidemiological characteristics and prognostic factors highlighted in this study set the stage for developing more personalized and effective clinical strategies for PB.

## Data Availability

Raw data used in analyses is available in SEER database (http://seer.cancer.gov/seerstat).
